# Reliability and Validity of the Greek QLQ-C30 and QLQ-MY20 for Measuring Quality of Life in Patients with Multiple Myeloma

**DOI:** 10.1100/2012/842867

**Published:** 2012-08-02

**Authors:** Nick Kontodimopoulos, Alexandros Samartzis, Angelos A. Papadopoulos, Dimitris Niakas

**Affiliations:** ^1^School of Social Sciences, Hellenic Open University, Bouboulinas 57-59, 26222 Patras, Greece; ^2^Department of Nuclear Medicine, Evangelismos General Hospital, Ipsilantou 45-47, 10676 Athens, Greece

## Abstract

*Objectives*. The aim of this study was to assess the psychometric properties of the Greek EORTC QLQ-C30 and QLQ-MY20 instruments. *Method*. A sample of myeloma patients (*N* = 89) from two tertiary hospitals were surveyed with the QLQ-C30, QLQ-MY20 and various demographic and disease related questions. The previously validated Greek SF-36 instrument was used as a “gold standard” for health-related quality of life (HRQoL) comparisons. Hypothesized scale structure, internal consistency reliability (Cronbach's alpha) and various forms of construct validity (convergent, discriminative, concurrent and known-groups) were assessed. *Results*. Multitrait scaling confirmed scale structure of the QLQ-C30 and QLQ-MY20, with good item convergence (96% and 72%) and discrimination (78% and 58%) rates. Cronbach's **α** was >0.70 for all but one scale (cognitive functioning). Spearman's correlations between similar QLQ-C30 and SF-36 scales ranged between 0.35–0.80 (*P* < 0.001). Expected interscale correlations and known-groups comparisons supported construct validity. QLQ-MY20 scales showed comparatively lower correlations with QLQ-C30 functional scales, and higher correlations with conceptually related symptom scales. *Conclusions*. The observed psychometric properties of the two instruments imply suitability for assessing myeloma HRQoL in Greece. Future studies should focus on generalizability of the results, as well as on specific issues such as longitudinal validity and responsiveness.

## 1. Introduction

Multiple myeloma (MM) is the second most prevalent blood cancer (13%), after non-Hodgkin's lymphoma, and represents approximately 1% of all cancers [1]. The average age of onset is 70 years and it affects slightly more men than women. The precise etiology of MM has not yet been established, although the possible role of environmental and/or occupational factors [2] and low socioeconomic status [3] has been suggested. Myeloma is generally thought to be incurable with a median survival of five years [4], which might be prolonged in younger patients, due to less comorbidity. Recent years have seen advances in treatment with steroids, chemotherapy, radiation, thalidomide, stem cell transplants, and newer drugs such as lenalidomide and bortezomib, all of which aim to control the disease and prolong survival. However, these palliative and life-prolonging treatments are associated with various side effects which impair health-related quality of life (HRQoL). Typically affected dimensions are physical functioning, fatigue, and pain [5].

Today, HRQoL is considered an important endpoint in cancer clinical trials, and a better understanding of it may lead to enhanced care of cancer patients in the future [6]. However, in haematological research the number of studies reporting HRQoL, either as a primary or a secondary endpoint, is low compared to studies involving other cancer patients [7]. According to a recent review of  MM-randomized controlled trials also assessing HRQoL (among other endpoints), the methodology of collecting and reporting HRQoL must be improved before results might actually influence clinical decision making [8]. The same review identified the European Organization for Research and Treatment of Cancer Core Quality of Life Questionnaire (EORTC QLQ-C30) [9] as the most frequently used instrument for measuring patient-reported HRQoL in MM, as it has been previously demonstrated to be reliable and valid in this particular patient group [10].

The EORTC has developed a myeloma module referred to as QLQ-MY20, to be administered alongside the core QLQ-C30 [11]. The module has been validated and shown to be measuring additional aspects of HRQOL, such as body image and future perspective [12]. Both instruments have been translated into Greek according to procedures reported elsewhere [13]. In particular, the QLQ-C30 has gained popularity in Greece, in part due to recent studies having demonstrated its psychometric properties in heterogeneous [14, 15] and homogenous [16] samples. We are, however, unaware of any Greek study having tested reliability and validity of the QLQ-C30 on MM patients. Moreover, despite being the only myeloma-specific instrument available in Greek, the QLQ-MY20 has not had its psychometric properties tested. In this context, the present validation is expected to increase confidence on future results obtained from the Greek QLQ-C30 and QLQ-MY20 and to make a contribution to the existing international body of knowledge on the subject.

## 2. Methodology

### 2.1. Instruments

The EORTC QLQ-C30 consists of 30 items and raw patient responses are transformed to produce 0–100 scores on five functional scales, nine symptom scales, and a scale representing global quality of life. Higher functional scale scores indicate better HRQoL, whereas higher symptom scale/item scores indicate higher level of symptoms [17]. The scales have undergone psychometric testing, based on classical test theory, which has yielded favorable results. The QLQ-MY20 is a 20-item myeloma module intended for use among patients varying in disease stage and treatment modality. The hypothesized scale structure consists of two symptom scales (disease symptoms and side effects of treatment), one-function scale (future perspective) and one single item (body image). The scoring approach is identical in principle to that for the function and symptom scale/items of the QLQ-C30, that is, a higher score on the symptom scales reflects a higher level of symptoms, whereas a higher score on future perspective, social support, and body image indicates good functioning or support. The validated Greek version [18, 19] of the generic SF-36 instrument [20] was used as the “gold standard” for HRQoL assessment in this study.

### 2.2. Patients and Data Collection

The data were collected between November 2009 and April 2010. Patients were recruited from two tertiary care hospitals in Athens: Evangelismos General Hospital and Alexandra General Hospital. It is noteworthy that the latter is a reference point in Greece for treating MM and has participated in many international research protocols [21–24]. Subjects were approached during a scheduled visit to one of the hospitals. None were suffering from cancer metastases or severe comorbid conditions which could further compromise HRQoL. The QLQ-C30, QLQ-MY20, and SF-36 were administered for self-completion followed by treatment-related and sociodemographic questions, in the presence of a trained study researcher to minimize missing data. Completion time was 20–30 minutes and all participants provided informed consent. The study was ethically approved by each Hospital's Review Board.

### 2.3. Analysis

Percentages of ceiling and floor scores were calculated as an indication of the instruments' ability to distinguish between subjects at the top and bottom parts of the scale, and a 50% threshold was adopted for both [25]. Scale internal consistency reliability was assessed via Cronbach's alpha and the 0.70 standard for group-level comparisons was adopted [26]. Item internal consistency, which is substantial when correlation between an item and its hypothesized scale (corrected for overlap) is >0.40, and item discriminant validity which is successful when correlation between an item and its own scale is significantly higher (>2 standard errors) than with other scales was used to examine the scale structure [27].

Spearman's correlations between QLQ-C30 and SF-36 scales were used to assess convergent construct validity. Based on the literature [28, 29], scales measuring similar HRQoL dimensions were hypothesized to be strongly correlated (>0.50 [30]). Construct validity was also examined via the interscale correlations between QLQ-MY20 and QLQ-C30, assuming that conceptually related scales would correlate substantially (>0.40), and that scales with less in common would show lower correlations [9, 31]. Each SF-36 scale was separately regressed against the QLQ-C30 and QLQ-MY20 scales to identify aspects of the disease-specific instruments more closely linked to general HRQoL [32]. Bonferroni correction has been used to adjust all *P* values to account for multiple testing bias.

Tests of “known groups” validity were performed by comparing scale scores across groups known to differ by sociodemographic data and clinical indicators. According to the literature, men were expected to report better functioning on all QLQ-C30 scales, higher global QOL, and fewer symptoms than women [33]. A decrease of functioning scores with increasing age was expected for both genders, whereas a stem cell transplantation should be associated with better scores. Mann-Whitney and Kruskal-Wallis tests were used to examine group differences. Age was treated as a continuous variable by examining its correlation to scale scores. Regarding the QLQ-MY20, we are unaware of any published stratification of scores according to gender and age and thus can only hypothesize that the direction of the differences should assimilate those of the QLQ-C30. All statistical analyses were performed with SPSS version 15.0.

## 3. Results

Ninety patients from the two participating centers (33 and 57, resp.) were interviewed and only one patient refused to participate (response rate 98.9%). The two subgroups did not differ significantly in gender and age-group distribution, or in mean QLQ-C30 and QLQ-MY20 scores, implying that treatment center would not confound the results. The majority of the participants (*N* = 89) were males (56.2%) and diagnosed with MM 3.9 ± 2.3 (mean ± SD) years ago on average. Their age ranged between 36 and 87 (mean 64.2 ± 11.3, median 66), and most patients were married (80.9%). At diagnosis, 37 (41.6%) patients were classified as stage I, 29 (32.6%) as stage II, and 17 (19.1%) as stage III, and according to the criteria set by International Myeloma Working Group [34] whereas for 6 (6.7%) data were not available. Forty-one (46.1%) patients had previously undergone an autologous stem cell transplantation, 44 (49.4%) had not, whereas for 4 patients (4.4%) data were again unavailable.

Data on central tendency, variability, and reliability of QLQ-C30 and QLQ-MY20 scales are presented in Table 1. Five symptom scales/items of the core QLQ-C30, that is, nausea/vomiting, appetite loss, constipation, diarrhea, and financial difficulties suffered from high (>50%) floor scores, implying a lack of these symptoms in this sample, but also suggest an underlying reduced discriminative ability. Conversely, no ceiling effects were observed on the core instrument despite three scales being close to the threshold value (role, cognitive, and social functioning). As for the myeloma-specific module, only one ceiling effect was observed, regarding the body image scale. Throughout both instruments, only one scale (cognitive functioning) did not meet the 0.70 internal consistency criterion.

Significantly higher item-scale correlations between items and their hypothesized scales than with competing scales were observed (Table 2). The 0.40 item-scale correlation criterion was satisfied, confirming item convergence, in 23/24 (95.8%) and 13/18 (72.2%) tests for QLQ-C30 and QLQ-MY20 scales, respectively. Accordingly, item discrimination was successful in 149/192 (77.6%) and 21/36 (58.3%) of the respective tests. The most obvious scaling failures occurred in the cognitive functioning and treatment side effects scales. Seven out of eight hypothesized strong correlations (>0.50) between pairs of QLQ-C30 and SF-36 scales measuring similar dimensions were confirmed (Table 3). The only expected correlation between scales not confirmed was between general health scale of the SF-36 and global health status of the QLQ-C30 (*ρ* = 0.35). Noteworthy, strong correlations were observed between the QLQ-C30 and SF-36 physical functioning (*ρ* = 0.80) and pain scales (*ρ* = −0.73). All hypothesized strong correlations were statistically significant (*P* < 0.001).

QLQ-MY20 scales expectedly showed comparatively low correlations (Table 4) with QLQ-C30 functioning scales (Spearman's *ρ* < 0.40) in 11 out of 18 comparisons (61.1%). On the other hand, expected strong correlations were confirmed between conceptually related QLQ-C30 symptom scales and QLQ-MY20 scales, for example, disease symptoms and pain (*ρ* = 0.74), side effects and fatigue (*ρ* = 0.60) and side effects and nausea/vomiting (*ρ* = 0.42). Interscale correlations between scales of the myeloma-specific module showed a weak correlation between future perspectives and disease symptoms (*ρ* = −0.24).

Linear regression identified those QLQ-C30 and QLQ-MY20 scales most closely associated with specific domains of HRQoL (represented by SF-36 scales). In the core instrument, only one scale (nausea/vomiting) and two symptoms items (constipation and financial difficulties) were not significant predictors of any SF-36 scale. Explanatory power was satisfactory for most models, with the physical functioning (68.7%) and mental health (61.8%) models demonstrating the highest. These results are presented in Table 5 in which only significant predictors are shown. Regarding the myeloma module, all scales were significantly related to at least one SF-36 scale, and once again the physical functioning and mental health models showed the highest explanatory power (32.4% and 45.3%, resp.).

Regarding the ability of the instruments to distinguish between known groups, men reported better functioning and fewer symptoms than women on all scales (*P* < 0.05 or better). The differences were in the expected directions for the single-item symptom scales as well, although differences were not significant. Men also outscored women (*P* < 0.01 or better) on the QLQ-MY20 disease symptoms, side effects, and body image scales. Increasing age was associated with decreased physical and cognitive functioning, more constipation and disease symptoms (*P* < 0.05). A previous transplant generated better scores on all scales however the only significant difference was in the QLQ-C30 physical functioning scale (*P* < 0.05).

## 4. Discussion

The present study aimed to assess the reliability and validity of the Greek versions of the QLQ-C30 and QLQ-MY20 and, in doing so, to increase confidence in results from future studies in Greece using these instruments. The suitability of the EORTC QLQ-C30 has been tested in MM patients [10, 35], but there are no other validated myeloma-specific quality-of-life questionnaires. Interestingly, a recent review of myeloma RCTs published after 1990, in which HRQoL assessment was included [8], identified only one study employing the QLQ-MY20 module [36]. According to EORTC data [37], the QLQ-MY20 module has been translated into more than 40 languages; however, we are unaware of any published validation studies regarding non-English versions of the instrument. Findings from the current study help fill an existing gap in Greece and contribute to the international literature on the subject.

The mean QLQ-C30 scores obtained from this sample of Greek patients are comparable to the EORTC reference values from a large sample of patients (*N* = 944) from a number of countries participating in various EORTC consortia [38]. The overall results for the psychometric properties of the two instruments, which was the main issue in this study, were satisfactory. Generally low floor and ceiling effects were observed, implying good discriminative ability and, perhaps, good responsiveness as well, although the latter could not be assessed in the present study due to its cross-sectional design. Although the high floor effects on five symptom scales of the core instrument might be threatening this assertion, QLQ-C30 reference values reported by the EORTC show quite similar results [38].

Internal consistency reliability was acceptable, thus providing evidence that each scale is measuring a distinct construct. The only noteworthy exception was the QLQ-C30 two-item cognitive functioning scale (*a* = 0.568), which has demonstrated exceptionally low reliability (*a* < 0.70) in QLQ-C30 validation studies in Greece [16] and elsewhere as well [29, 39–41]. The two aspects of cognitive functioning assessed, that is, concentration and memory, are not necessarily strongly associated with each other. For example, a patient who cannot concentrate well due to pain or fatigue may actually have good memory [29]. However, poor internal consistency reliability may be balanced by clinical relevance, which is an equally important consideration in HRQoL instrument development [42].

Good results for multitrait scaling confirmed the hypothesized scale structure, implying that the translation of the items and the response choices are appropriate and that scale scores derived from the Greek version could contribute to cross-cultural comparisons. Cognitive functioning and treatment side effects were the only exceptions to overall scaling success for the QLQ-C30 and QLQ-MY20, respectively. The former was expected as a result of the scale's low internal consistency reliability mentioned previously. On the other hand, most of the items of the side effects scale showed strong correlations with the disease symptoms scale as well, and this explains its compromised item-scale discriminant validity. Expectedly, correlations between QLQ-MY20 scales and QLQ-C30 functional scales were comparatively lower than those among QLQ-MY20 and conceptually related QLQ-C30 symptom scales. Interscale correlations between scales of the myeloma-specific model showed strong correlation between disease symptoms and treatment side effects (*ρ* = 0.50, *P* < 0.001), which might be implying some overlap in the constructs measured.

Criterion-related construct validity was supported by confirmation of hypothesized strong correlations between QLQ-C30 and SF-36 scales measuring similar dimensions. Furthermore, most QLQ-C30 scales correlated more strongly with SF-36 scales measuring similar HRQoL dimensions than with those measuring different ones, providing evidence that scale captions imply conceptual similarities present on individual item levels. An exception was observed for the QLQ-C30 global QoL scale which demonstrated a stronger correlation with the SF-36 vitality scale than with the general health scale. Similar results have been reported elsewhere [16–29]. Possible explanations might be that the two instruments operationalize the HRQoL construct differently [43], or perhaps artefacts from the translation from English to Greek.

The OLS regression models showed relatively high proportions of HRQoL variance explained. The QLQ-C30 physical functioning scale was a significant predictor of four SF-36 scales, namely physical functioning, pain, general health, and vitality. Similarly, all QLQ-MY20 scales were significant predictors of at least two SF-36 scales, with body image contributing to five. These results provide evidence that the Greek versions of the instruments are indeed measuring standard quality of life aspects. Although disease symptoms are usually uncontrollable, aspects such as body image and future perspectives are perhaps easier to manipulate, and a systematic approach to their improvement may prove beneficial in terms of HRQOL for the MM population. Portions of variability of each SF-36 scale remained unexplained, since other demographic or health-related variables were not used in the regression models, and this could trigger further research.

Known-groups comparisons (quantitative results not shown for parsimony) generated expected results in terms of score differences and the direction of these differences. On the other hand, not meeting the criterion of statistical significance in some known-groups comparisons might be an artifact of confounding clinical and treatment factors which could not be controlled for, mostly due to sample size [16]. Comparisons of HRQoL according to disease stage were not performed because, unfortunately, we had access to the relevant data only at diagnosis (up to four years back for some patients) and it is likely that stage has changed. It should be noted that all other variables are contemporaneous with questionnaire administration.

The nature and design of this relatively small, cross-sectional study obviously limit its generalizability, but the work lays the foundation for future studies. Although internal consistency reliability and cross-sectional construct validity of the Greek QLQ-C30 and QLQ-MY20 were satisfactorily demonstrated, test-retest reliability and longitudinal construct validity and responsiveness were not addressed. These properties are particularly important as cancer patients' health status changes. Thus, a longitudinal study design could be considered to overcome these limitations. Overall, the psychometric properties of the Greek version were similar to the original English version for the QLQ-MY20 and other language versions for the QLQ-C30 [12, 29, 31, 39–42, 44]. In an attempt by physicians to choose better therapy protocols and adopt better care strategies for patients, these two instruments appear to be suitable for HRQoL measurement in Greek myeloma patients.

## Figures and Tables

**Table 1:** Central tendency, variability, and reliability of the QLQ-C30 and QLQ-MY20 scales.

	No. of Items	Mean (SD)	95% CI	Median	Floor (%)	Ceiling (%)	Reliability^a^
QLQ-C30							
Global health status/QoL	2	62.6 (21.6)	58.1–67.2	66.7	0.0 (3.4)	100.0 (5.6)	0.92
Functional scales							
Physical functioning	5	64.0 (22.3)	59.3–68.7	66.7	0.0 (1.1)	100.0 (4.5)	0.80
Role functioning	2	68.9 (33.9)	61.8–76.1	66.7	0.0 (10.1)	100.0 (41.6)	0.90
Emotional functioning	4	71.5 (25.1)	66.3–76.8	75.0	0.0 (2.2)	100.0 (12.4)	0.84
Cognitive functioning	2	81.7 (23.8)	76.6–86.7	83.3	0.0 (1.1)	100.0 (48.3)	0.57
Social functioning	2	79.0 (25.2)	73.7–84.3	83.3	16.7 (4.5)	100.0 (46.1)	0.77
Symptom scales/items							
Fatigue	3	41.3 (27.6)	35.5–47.1	33.3	0.0 (10.1)	100.0 (4.5)	0.89
Nausea and vomiting	2	8.2 (20.4)	3.9–12.5	0.0	0.0 (82.0)	100.0 (1.1)	0.74
Pain	2	29.8 (27.8)	23.9–35.6	33.3	0.0 (30.3)	100.0 (3.4)	0.80
Dyspnoea	1	34.5 (32.4)	27.6–41.3	33.3	0.0 (34.8)	100.0 (10.1)	—
Insomnia	1	32.6 (36.2)	25.0–40.2	33.3	0.0 (46.1)	100.0 (13.5)	—
Appetite loss	1	13.9 (29.2)	7.7–20.0	0.0	0.0 (77.5)	100.0 (6.7)	—
Constipation	1	22.9 (32.8)	15.9–29.8	0.0	0.0 (58.4)	100.0 (10.1)	—
Diarrhea	1	9.4 (18.1)	5.6–13.2	0.0	0.0 (75.3)	100.0 (1.1)	—
Financial difficulties	1	12.0 (22.6)	7.2–16.8	0.0	0.0 (74.2)	100.0 (1.1)	—

QLQ-MY20							
Disease symptoms	6	20.2 (19.8)	16.0–24.3	16.7	0.0 (25.8)	83.3 (1.1)	0.77
Side effects of treatment	10	20.1 (15.5)	16.8–23.3	18.5	0.0 (5.6)	70.4 (1.1)	0.72
Body image	1	72.3 (34.5)	65.0–79.6	100.0	0.0 (9.0)	100.0 (53.9)	—
Future perspective	3	54.6 (29.8)	48.3–60.8	55.6	0.0 (7.9)	100.0 (10.1)	0.80

^a^Cronbach's alpha.

**Table 2:** Summary results of scaling assumption tests.

	*N* ^ a^	Item-internal consistency		Item-discriminant validity	
		Range of correlations^b^	# Success/total^c^	Range of correlations^d^	# Success/total^e^
QLQ-C30					
Global health status/QoL	2	0.85	2/2	0.34–0.59	16/16
Functional scales					
Physical functioning	5	0.35–0.73	4/5	0.17–0.55	27/40
Role functioning	2	0.82	2/2	0.27–0.64	15/16
Emotional functioning	4	0.62–0.74	4/4	0.21–0.68	29/32
Cognitive functioning	2	0.40	2/2	0.10–0.47	2/16
Social functioning	2	0.65	2/2	0.22–0.51	10/16
Symptom scales/items					
Fatigue	3	0.75–0.83	3/3	0.27–0.57	22/24
Nausea and vomiting	2	0.66	2/2	0.08–0.41	16/16
Pain	2	0.67	2/2	0.14–0.56	12/16

QLQ-MY20					
Disease symptoms	6	0.18–0.68	5/6	0.01–0.51	9/12
Side effects of treatment	9^f^	0.23–0.57	5/9	0.03–0.43	6/18
Future perspective	3	0.60–0.69	3/3	0.16–0.38	6/6

^a^Number of items and number of item-internal consistency tests per scale (single-item scales excluded).

^b^Range of correlations between items and hypothesized scale corrected for overlap.

^c^Number of correlations exceeding the 0.40 standard/total number of correlations.

^d^Range of correlations between items and other scales.

^e^Number of successful discriminant validity tests/total number of discriminant validity tests.

^f^Item no.42 concerned only 10% of the sample suffering hair loss and was omitted from this analysis.

**Table 3:** Spearman rank correlations between EORTC QLQ-C30 and SF-36 scales.

QLQ-C30 scales	SF-36 scales							
	PF	RP	BP	GH	VT	SF	RE	MH
Global health status	0.58^∗∗∗^	0.27	0.52^∗∗∗^	**0.35** ^ ∗^	0.59^∗∗∗^	0.48^∗∗∗^	0.31	0.51^∗∗∗^
Physical functioning	**0.80** ^ ∗∗∗^	0.51^∗∗∗^	0.51^∗∗∗^	0.41^∗∗∗^	0.71^∗∗∗^	0.54^∗∗∗^	0.26	0.49^∗∗∗^
Role functioning	0.54^∗∗∗^	**0.51** ^ ∗∗∗^	0.47^∗∗∗^	0.24	0.57^∗∗∗^	0.47^∗∗∗^	**0.50** ^ ∗∗∗^	0.36^∗^
Emotional functioning	0.39^∗∗^	0.26	0.44^∗∗∗^	0.35^∗^	0.54^∗∗∗^	0.48^∗∗∗^	0.41^∗∗^	**0.71** ^ ∗∗∗^
Social functioning	0.55^∗∗∗^	0.31	0.49^∗∗∗^	0.32	0.48^∗∗∗^	**0.59** ^ ∗∗∗^	0.27	0.39^∗∗^
Fatigue	−0.52^∗∗∗^	−0.42^∗∗^	−0.45^∗∗∗^	−0.44^∗∗^	**−0.64** ^ ∗∗∗^	−0.47^∗∗∗^	−0.31	−0.61^∗∗∗^
Pain	−0.50^∗∗∗^	−0.37^∗^	**−0.73** ^ ∗∗∗^	−0.28	−0.42^∗∗^	−0.29^∗^	−0.21	−0.33

Notes: bold numbers indicate correlations hypothesized to be moderate or strong. Negative correlations are an artifact of the scoring procedures.

PF: physical functioning, RP: role physical, BP: bodily pain, GH: general health, VT: vitality, SF: social functioning, RE: role emotional, and MH: mental health.

*P < 0.05, **P < 0.01, and ***P < 0.001, with Bonferroni adjustment for multiple testing.

**Table 4:** Interscale correlations among QLQ-MY20 scales and with QLQ-C30 scales.

QLQ-MY20 scales	Disease symptoms	Side effects	Future perspectives
Disease symptoms	—	—	—
Side effects	0.50^∗∗∗^	—	—
Future perspectives	−0.24	−0.45^∗∗∗^	—

QLQ-C30 scales			
Global health status	−0.33^∗^	−0.50^∗∗∗^	0.33^∗^
Physical functioning	−0.48^∗∗∗^	−0.62^∗∗∗^	0.38^∗∗^
Role functioning	−0.24	−0.44^∗∗∗^	0.27
Emotional functioning	−0.27	−0.54^∗∗∗^	0.60^∗∗∗^
Cognitive functioning	0.26	−0.43^∗∗∗^	0.20
Social functioning	−0.33	−0.44^∗∗∗^	0.27
Fatigue	0.35^∗^	0.60^∗∗∗^	−0.45^∗∗∗^
Nausea and vomiting	0.20	0.42^∗∗∗^	−0.14
Pain	0.74^∗∗∗^	0.48^∗∗∗^	−0.20

Negative correlations are an artifact of the scoring procedure.

*P < 0.05, **P < 0.01, and ***P < 0.001, with Bonferroni adjustment.

**Table 5:** Multivariate analyses (*B* coefficient ^(p-sig.)^).

Dependent variable predictors	Dependent Variable							
	PF	RP	BP	GH	VT	SF	RE	MH
QLQ-C30								
Global health status	0.22	—	0.38^∗^	—	0.22	—	—	—
Physical functioning	0.83^∗∗∗^	0.56	—	0.23	0.34^∗∗∗^	—	—	—
Role functioning	—	0.36	—	—	—	0.20	0.49^∗∗^	—
Emotional functioning	—	—	—	—	—	0.24	—	0.48^∗∗∗^
Cognitive functioning	—	—	—	—	—	—	0.47	—
Social functioning	—	—	—	—	—	0.45^∗∗^	—	—
Fatigue	—	—	—	−0.20	−0.20^∗∗^	—	—	—
Nausea and vomiting	—	—	—	—	—	—	—	—
Pain	—	—	−0.67^∗∗∗^	—	—	—	—	—
Dyspnoea	−0.13	—	—	—	—	—	—	—
Insomnia	—	—	—	—	—	—	—	−0.11
Appetite loss	—	—	—	—	—	—	—	−0.19^∗^
Constipation	—	—	—	—	—	—	—	—
Diarrhea	—	—	—	—	−0.17	—	—	—
Financial difficulties	—	—	—	—	—	—	—	—
Constant	−13.46	−25.09	54.26	41.46	24.60	−1.00	−13.02	32.29
Adjusted *R* ^2^	**0.687**	**0.290**	**0.601**	**0.195**	**0.588**	**0.421**	**0.282**	**0.618**

QLQ-MY20								
Disease symptoms	−0.40^∗^	—	−0.90^∗∗∗^	−0.28^∗^	—	—	—	—
Side effects of treatment	−0.68^∗∗^	—	—	—	−0.66^∗∗∗^	−0.47	—	−0.50^∗∗∗^
Body image	—	0.39^∗∗^	—	—	0.13	0.25^∗^	0.34^∗^	0.15
Future perspective	—	—	—	0.23^∗∗^	—	—	—	0.21^∗∗^
Constant	70.51	7.21	75.96	40.88	54.96	56.18	33.90	48.27
Adjusted *R* ^2^	**0.324**	**0.101**	**0.330**	**0.205**	**0.390**	**0.205**	**0.069**	**0.453**

PF: physical functioning, RP: role-physical, BP: bodily pain, GH: general health, VT: vitality, SF: social functioning, RE: role emotional, and MH: mental health.

*P < 0.05, **P < 0.01, and ***P < 0.001, according to OLS regression with stepwise selection and Bonferroni adjustment for multiple testing.

## References

[1] Raab MS, Podar K, Breitkreutz I, Richardson PG, Anderson KC (2009). Multiple myeloma. *The Lancet*.

[2] Fritschi L, Siemiatycki J (1996). Lymphoma, myeloma and occupation: results of a case-control study. *International Journal of Cancer*.

[3] Koessel SL, Theis MK, Vaughan TL (1996). Socioeconomic status and the incidence of multiple myeloma. *Epidemiology*.

[4] Sirohi B, Powles R (2006). Epidemiology and outcomes research for MGUS, myeloma and amyloidosis. *European Journal of Cancer*.

[5] Gulbrandsen N, Hjermstad MJ, Wisløff F (2004). Interpretation of quality of life scores in multiple myeloma by comparison with a reference population and assessment of the clinical importance of score differences. *European Journal of Haematology*.

[6] Bottomley A, Quinten C, Coens C (2009). Making better use of existing cancer data: patient reported outcomes and behavioural evidence: a new international initiative. *European Journal of Cancer Care*.

[7] Efficace F, Kemmler G, Vignetti M, Mandelli F, Molica S, Holzner B (2008). Health-related quality of life assessment and reported outcomes in leukaemia randomised controlled trials—a systematic review to evaluate the added value in supporting clinical decision making. *European Journal of Cancer*.

[8] Kvam AK, Fayers P, Hjermstad M, Gulbrandsen N, Wisloff F (2009). Health-related quality of life assessment in randomised controlled trials in multiple myeloma: a critical review of methodology and impact on treatment recommendations. *European Journal of Haematology*.

[9] Aaronson NK, Ahmedzai S, Bergman B (1993). The European organization for research and treatment of cancer QLQ-C30: a quality-of-life instrument for use in international clinical trials in oncology. *Journal of the National Cancer Institute*.

[10] Wisloff F, Eika S, Hippe E (1996). Measurement of health-related quality of life in multiple myeloma. *British Journal of Haematology*.

[11] Stead ML, Brown JM, Velikova G (1999). Development of an EORTC questionnaire module to be used in health- related quality-of-life assessment for patients with multiple myeloma. *British Journal of Haematology*.

[12] Cocks K, Cohen D, Wisløff F (2007). An international field study of the reliability and validity of a disease-specific questionnaire module (the QLQ-MY20) in assessing the quality of life of patients with multiple myeloma. *European Journal of Cancer*.

[13] Dewolf L, Koller M, Velikova G (2009). *EORTC Quality of Life Group Translation Procedure*.

[14] Mystakidou K, Tsilika E, Parpa E, Kalaidopoulou O, Smyrniotis V, Vlahos L (2001). The EORTC core quality of life questionnaire (QLQ-C30, version 3.0) In terminally ill cancer patients under palliative care: validity and reliability in a hellenic sample. *International Journal of Cancer*.

[15] Koukouli S, Stamou A, Alegakis A, Georgoulias V, Samonis G (2009). Psychometric properties of the QLQ-C30 (version 3.0) in a sample of ambulatory Cretan cancer patients. *European Journal of Cancer Care*.

[16] Kontodimopoulos N, Ntinoulis K, Niakas D (2011). Validity of the Greek EORTC QLQ-C30 and QLQ-BR23 for measuring health-related quality of life in breast cancer patients. *European Journal of Cancer Care*.

[17] Fayers P, Aaronson N, Bjordal K, Groenvold M, Curran D, Bottomley A (2001). *EORTC QLQ-C30 Scoring Manual EORTC Quality of Life Group*.

[18] Pappa E, Kontodimopoulos N, Niakas D (2005). Validating and norming of the Greek SF-36 Health Survey. *Quality of Life Research*.

[19] Anagnostopoulos F, Niakas D, Pappa E (2005). Construct validation of the Greek SF-36 Health Survey. *Quality of Life Research*.

[20] Ware JE, Sherbourne CD (1992). The MOS 36-item short-form health survey (SF-36). I. Conceptual framework and item selection. *Medical Care*.

[21] Dimopoulos M, Spencer A, Attal M (2007). Lenalidomide plus dexamethasone for relapsed or refractory multiple myeloma. *New England Journal of Medicine*.

[22] Dimopoulos M, Terpos E, Comenzo RL (2009). International myeloma working group consensus statement and guidelines regarding the current role of imaging techniques in the diagnosis and monitoring of multiple Myeloma. *Leukemia*.

[23] Palumbo A, Sezer O, Kyle R (2009). International Myeloma Working Group guidelines for the management of multiple myeloma patients ineligible for standard high-dose chemotherapy with autologous stem cell transplantation. *Leukemia*.

[24] Terpos E, Dimopoulos MA, Sezer O (2010). The use of biochemical markers of bone remodeling in multiple myeloma: a report of the International Myeloma Working Group. *Leukemia*.

[25] Wan-Po AL (1998). *Dictionary of Evidence-Based Medicine*.

[26] Nunnaly JC, Bernstein IR (1994). *Psychometric Theory*.

[27] Ware JE, Gandek B (1998). Methods for testing data quality, scaling assumptions, and reliability: the IQOLA Project approach. *Journal of Clinical Epidemiology*.

[28] Apolone G, Filiberti A, Cifani S, Ruggiata R, Mosconi P (1998). Evaluation of the EORTC QLQ-C30 questionnaire: a comparison with SF-36 Health Survey in a cohort of Italian long-survival cancer patients. *Annals of Oncology*.

[29] Luo N, Fones CSL, Lim SE, Xie F, Thumboo J, Li SC (2005). The European organization for research and treatment of cancer quality of life questionnaire (EORTC QLQ-C30): validation of English version in Singapore. *Quality of Life Research*.

[30] Cohen J (1992). A power primer. *Psychological Bulletin*.

[31] Jayasekara H, Rajapaksa LC, Brandberg Y (2008). Measuring breast cancer-specific health-related quality of life in South Asia: psychometric properties of the Sinhala version of the EORTC QLQ-BR23. *Quality of Life Research*.

[32] Kontodimopoulos N, Niakas D (2005). Determining the basic psychometric properties of the Greek KDQOL-SF. *Quality of Life Research*.

[33] Hjermstad MJ, Fayers PM, Bjordal K, Kaasa S (1998). Health-related quality of life in the general Norwegian population assessed by the European organization for research and treatment of cancer core quality-of-life questionnaire: the QLQ=C30 (+3). *Journal of Clinical Oncology*.

[34] Greipp PR, Miguel JS, Dune BGM (2005). International staging system for multiple myeloma. *Journal of Clinical Oncology*.

[35] Wisløff F, Hjorth M (1997). Health-related quality of life assessed before and during chemotherapy predicts for survival in multiple myeloma. *British Journal of Haematology*.

[36] Sirohi B, Powles R, Lawrence D (2007). An open, randomized, controlled, phase II, single centre, two-period cross-over study to compare the quality of life and toxicity experienced on PEG interferon with interferon-*α*2b in patients with multiple myeloma maintained on a steady dose of interferon-*α*2b. *Annals of Oncology*.

[37] European Organization for Research and Treatment of Cancer (EORTC). http://groups.eortc.be/qol/index.htm.

[38] Scott NW, Fayers PM, Aaronson NK (2008). *EORTC QLQ-C30 Reference Values*.

[39] Chie WC, Chang KJ, Huang CS, Kuo WH (2003). Quality of life of breast cancer patients in Taiwan: validation of the Taiwan Chinese version of the EORTC QLQ-C30 and EORTC QLQ-BR23. *Psycho-Oncology*.

[40] Wan C, Tang X, Tu XM (2007). Psychometric properties of the simplified Chinese version of the EORTC QLQ-BR53 for measuring quality of life for breast cancer patients. *Breast Cancer Research and Treatment*.

[41] Awad MA, Denic S, El Taji H (2008). Validation of the European organization for research and treatment of cancer quality of life questionnaires for Arabic-speaking populations. *Annals of the New York Academy of Sciences*.

[42] Yun YH, Bae SH, Kang IO (2004). Cross-cultural application of the Korean version of the European Organization for Research and Treatment of Cancer (EORTC) Breast-Cancer-Specific Quality of Life Questionnaire (EORTC QLQ-BR23). *Supportive Care in Cancer*.

[43] Kuenstner S, Langelotz C, Budach V, Possinger K, Krause B, Sezer O (2002). The comparability of quality of life scores: a multitrait multimethod analysis of the EORTC QLQ-C30, SF-36 and FLIC questionnaires. *European Journal of Cancer*.

[44] Montazeri A, Harirchi I, Vahdani M (2000). The EORTC breast cancer-specific quality of life questionnaire (EORTC QLQ-BR23): translation and validation study of the Iranian version. *Quality of Life Research*.

